# Developing ‘Smart’ Dairy Farming Responsive to Farmers and Consumer-Citizens: A Review

**DOI:** 10.3390/ani12030360

**Published:** 2022-02-02

**Authors:** Maeve Mary Henchion, Áine Regan, Marion Beecher, Áine MackenWalsh

**Affiliations:** 1 Department of Agrifood Business and Spatial Analysis, Rural Economy and Development Programme (REDP), Teagasc Ashtown Food Research Centre, D15 KN3K Dublin, Ireland; 2 Department of Agrifood Business and Spatial Analysis, REDP, Teagasc, Áras uí Mhaoilíosa, Athenry, Co., H65 R718 Galway, Ireland; aine.regan@teagasc.ie (Á.R.); aine.mackenwalsh@teagasc.ie (Á.M.); 3 Animal & Grassland Research and Innovation Centre, Teagasc, Moorepark, Fermoy Co., P61 C997 Cork, Ireland; marion.beecher@teagasc.ie

**Keywords:** responsible research and innovation, RRI, consumer, citizen, farmer, attitude, dairy, food system, innovation

## Abstract

**Simple Summary:**

Dairy production has evolved over many generations to be an important source of high-quality nutrition for a significant proportion of the global population. However, it needs to evolve further to ensure it contributes to sustainable diets. Technological innovation can be a key enabler. It is also the case however that innovation brings about significant change, and can introduce unexpected, unintended and undesirable consequences, which are experienced differently by different actors on the ground. Thus, a major challenge is turning good science and technology into positive and innovative outcomes for society in an equitable way. Drawing on concepts from Responsible Research and Innovation (anticipation, inclusion, reflexivity and responsiveness) and Food Systems thinking, the authors reviewed the academic literature to consider the perspectives of different actors relating to technologies on dairy farms. It considers ‘smart’ on-farm technologies at three key stages of the dairy production cycle—breeding, feeding and milking—through the lens of two actor groups. It considers the farmers who may(not) adopt such innovations and the consumer-citizens who will(not) purchase/accept the resultant on-farm practices and foods. It highlights some differences between and within these actor groups, but also identifies commonalities, including tensions, faced by both groups. Dairy production in the future, thus, is not only challenged with embracing advanced technologies, the process by which such technologies are designed and selected must also be ‘smart’.

**Abstract:**

Innovation has resulted in more dairy products being produced with less inputs than ever before. It has also affected how animals are raised, the structure of the sector and the nature of products produced. Not all impacts have been positive. As disruptive technologies—such as precision farming and robotics—herald significant change, it is timely to reflect on the perspectives of different actors on innovations within the sector. Drawing on a review of academic literature, this paper considers farmers’ and consumer-citizens’ perspectives; as expected, their diverse knowledge, interests and values surface a range of perspectives. To provide focus to the study, it examines technologies across three stages of the dairy production cycle: breeding, feeding and milking. It finds that consumer-citizen and farmer perspectives have been examined by researchers in several countries, using a variety of methods, across a range of technologies. It finds both areas of agreement and tension within and between consumer-citizen and producer cohorts. While differences in knowledge account for some variation, differences in values are also significant. The extent to which efforts can and should be put into addressing differences is raised as a point for reflection.

## 1. Introduction

The dairy sector has featured as an important component of the domestication of livestock for food purposes for about 360 human generations [1]. Advances in science and technology have supported the introduction of many on-farm innovations. Such innovations mean that the way animals are raised on farms has changed greatly over the past century [2] and such innovations have also had structural impacts on the sector through promoting specialization, mechanization and intensification [3]. Indeed the scale and intensity of livestock farming have increased significantly over the past 50 years [4]. This has had positive economic impacts including greater feed conversion efficiencies and increased yields, resulting in more dairy products being produced with less inputs than ever before, reduced manual working on farms, improvements in food safety and quality through the adoption of food safety and quality standards, and reduced costs of dairy products [5,6]. While the primary aim of such developments is to increase agricultural output and increase the availability of safe, affordable food, some developments have negatively affected other aspects of sustainability, including animal welfare [7], the environment, worker safety and rural economies [2,8]. For example, changes in farming practices associated with specialization and mechanization have resulted in reduced space allocation to animals, with some animals given very limited/no access to the outdoors. It has also resulted in animal husbandry practices that may be painful, for example castration and debudding, and practices that interfere with ‘natural’ behavior, e.g., removing calves from their dams [9]. These and other practices have provoked negative reactions, often based on ethical perspectives, from citizens in particular [9,10]. Intensification has also resulted in the increased use of (synthetic) inputs, with negative impacts on water and air quality, biodiversity and soil health. Such realities have led to questions about the sustainability of dairy production arising in relation not only to trade-offs across economic and environmental aspects of sustainability but also in relation to social (intersections between different groups or individuals) and temporal (intersections between different generations) trade-offs. Critical questions also arise in relation to the social acceptance of certain on-farm practices. Indeed, notwithstanding a progressive loss of connection between consumers and producers, leading to a lack of knowledge of animal production management approaches and practices by the general public [11], consumers are now increasingly questioning production systems and wanting to know more about how their food is produced [12]. While dairy production tends to have a relatively positive image amongst the public [9], being associated with animals grazing pasture and living in the countryside, practices in dairy farming are increasingly subject to public scrutiny [13].

Thus, the sustainable production of food, including dairy, to meet the demands of a growing global population cannot be assumed and it is increasingly recognized that there are “externalities” associated with food production that are often not sufficiently taken into account. These externalities are defined as both costs and benefits that arise due to the production or consumption of goods that are not reflected in the market price. Examples include ecological effects, water quality, resource depletion, greenhouse gas (GHG) emissions, animal welfare, cultural heritage, social costs associated with labor and public health effects [14]. Such thinking, and growing awareness of negative externalities in particular, has led to calls to transform the food system. A report by the Food and Land Use Coalition estimates that the global cost of these externalities is in the region of US$12 trillion a year, rising to $16 trillion by 2050 [15]. In this context, “top-down” government policy (dis)incentives are often relied upon to change behavior to influence the magnitude and distribution of costs and benefits. Taxes, regulations and financial incentives are some of the options available to governments. Another complementary option is to draw on the work of researchers in the behavioral sciences to use “bottom-up” approaches to change practices in industry [16], and to engage with stakeholders to clearly identify and address any potential unintended consequences as early as possible. A third option is to draw on advances in science and technology [17], including nature-based solutions, in an attempt to eliminate potential trade-offs. It is highly likely that responses to the call for food system transformation, particularly in the current context of the urgent need to address climate change, will lead to a new era of disruption and transformation in the agricultural sector, with significant consequences for the role of dairy farming, and the foods that consumers are willing to purchase. However, turning good science and technology into good practice on a dairy farm is a challenge. Reflecting a view that technologies are “socially shaped, co-created by their makers and users to perform roles that can change over time, and be different, for different groups of people“ [18] (p. 518), the term technology in this paper is used broadly and can encompass any or all of three layers—a physical object or artefact, a process or activity, and what people know as well as what they do [18,19] (p. 518).

The purpose of this research is to contribute to responsive and sustainable research and innovation practices within the dairy sector by surfacing the views of potential users and/or those who may be positively or negatively affected by such developments. Drawing on published academic literature that reports the results of qualitative and quantitative data collection methods designed to elicit such information, we identify some of the impacts that could arise with the introduction of key technologies in the dairy sector. We do so through the lens of two particular actor groups who have often tended to have little agency or power within the research and innovation process to date: farmers and consumer-citizens [20]. These actors represent opposite ends of the traditional value chain, thus potentially capturing some of the diversity of perspectives that may exist; previous research provides evidence of differences in perspectives between these cohorts on issues relating to agricultural production [21]. Through a series of specific technologies, across key stages of the dairy production cycle, we consider what we know to date about how farmers and consumer-citizens view and value aspects of these technologies. We do this to (1) illuminate the range of perspectives that may exist across these cohorts, (2) explore the extent to which differences exist within these cohorts, and (3) propose reasons for such differences. In this way, we can highlight knowledge gaps within the literature and suggest factors to be considered and strategies that could be adopted to support responsive and sustainable research and innovation practices. The paper starts by discussing Responsible Research and Innovation (RRI) and the centrality of ‘inclusion’ for societal responsiveness and food system transformation. It then argues for the consideration of on-farm technologies through the lenses of two key actor groups: the lens of farmers who may/may not adopt such innovations and the lens of consumers-citizens who will/will not purchase/accept the resultant foods. The following section considers technologies and associated practices at three key stages in the dairy production cycle—breeding, feeding (calf and cow) and milking—that are adopted on farms and/or are moving into widespread adoption. The paper concludes with a discussion on areas that researchers, extension workers and policy makers may wish to consider in developing more responsive and socially acceptable solutions to sustainability challenges facing the dairy sector.

## 2. Responsiveness to the Needs and Values of Farmers and Citizen-Consumers

New science and technologies are presenting opportunities to radically change how dairy food is produced and delivered to the market. They hold a lot of promise for ensuring a more ‘responsive’ dairy sector, a dairy sector that is more sustainable, safe and secure. Disruptive technologies and innovations such as precision farming, agro-genomics, robotics and digital traceability systems could be “ushering in a fourth agricultural revolution” [15], with some arguing that a transition is already occurring in the farming sector with the introduction of new advanced innovations and digital technologies [22]. In this argument, technological innovations are viewed as key enablers and drivers for supporting more sustainable, safe and secure farming and food systems [23], addressing some of the key challenges facing the dairy sector such as sustainability, climate change, food quality and nutrition, and animal health and welfare and “neutralising” some of the trade-offs that are implicit across different sustainability dimensions. However, such innovations also bring about significant change, and can introduce unexpected and unintended consequences in areas such as digital exclusion, unequal power relations, animal-human relationships, data governance, skill and identity loss [23,24].

Numerous scholars have called for more inclusive approaches to be used in research and innovation in agriculture broadly [25] and dairy farming specifically [20]. Arguments include the instrumental argument that understanding the perspectives of different actors can help to improve milk production practices on farm [26] and that societal preferences will continue to influence food production including dairy farming [5]. More normative arguments claim that unless there is an understanding of the needs and expectations of the public, the adoption of agricultural practices that are inconsistent with public expectations may undermine social sustainability [2].

### 2.1. Responsible Research and Innovation

Responsible Research and Innovation (RRI) is a framework championed at national levels and within the European Union to streamline the design and deployment of technology and innovation, and to ensure it is done in a manner that is reflective of and responsive to diverse societal needs and values [27,28]. The principles covered under RRI echo those from movements such as ELSA (Ethical, Legal and Social Aspects) and Public Understanding of Science. Four dimensions of RRI have been identified and described: anticipation, inclusion, reflexivity and responsiveness [27]. Anticipation encourages researchers and technology developers to explore possible impacts of their innovations and research, both the short-term and long-term consequences, and the positives and negatives. Inclusion or engagement highlights the importance of ensuring diverse actors have a voice during the innovation process from start to finish; allowing researchers and technology developers to be aware of diverse values, needs and concerns. Reflexivity encourages researchers and technology developers to consider their own personal values and conflicts of interest and the views of others. They are encouraged to reflect all the while on how these different values may converge or diverge, and what this may mean for developing and implementing new technologies and innovations. Finally, responsiveness occurs when researchers and technology developers make an active effort to respond to these inputs by altering their research or innovation trajectory accordingly.

The RRI framework is intended to support decision-makers driving technological innovation and to act as ‘a scaffold for raising, discussing and responding to questions of societal concern’ [29] (p. 245). There is no set or organized process for carrying out RRI exercises, although scholars argue that the lever of inclusion is central for operationalizing all dimensions [23]. One of the key principles of RRI is the need for inclusivity and engagement with diverse actors. Processes, such as collective experimentation and participatory design, or ‘co-design’, which facilitate dialogue and deliberation, are at the heart of this principle [30]. This anticipatory or upstream engagement enables conversations to happen between diverse actors before, and during, technology development and implementation to ensure that different values are accounted for and to avoid technocratic decision-making [29].

### 2.2. Inclusion of Citizen-Consumers and Farmers

In parallel with discussions on RRI, the need for inclusivity in how food systems transformation occurs is also emphasized. This is because various actors are likely to have different perspectives on the supports and incentives that should be provided, and from what source, to effect changes in the food system. The OECD [31] highlights likely differences, and thus potential sources of frictions, in terms of facts, expectations and values amongst different groups when seeking to develop policies and make decisions to support an effective food system, emphasizing the need to understand such different perspectives. In relation to the livestock sector, the organization indicates a role for independent advisory groups to establish the facts, but cautions that they are not always widely accepted by the public or stakeholders, and that there is a need to have a shared understanding of the facts amongst all stakeholders [31]. They suggest that the interests of different groups, including those with livelihoods at stake, can be surfaced through public consultations. In relation to values, they reference the importance of farmer identities and their sense of belonging to a rural community. They argue that “… making better policies for food systems not only requires a rigorous understanding of how the world is, but also a shared view of how the world should be” [31] (p. 10). Given the trade-offs that are inherent in developing sustainable food systems, they conclude that “a trade-off cannot be resolved on purely technical grounds, but involves an element of societal choice” [31] (p. 82).

As argued, there is a need for better inclusion of diverse actors’ values and needs in the decision-making surrounding new technologies and innovations in the dairy sector. Specifically, this paper argues that the views of two particular societal actors should be better considered: farmers, and consumer-citizens. Such groups are often presented in the literature as opposing or even in conflict. Moreover as they are likely to have different levels of knowledge and experience, different interests and draw on different value sets, it is likely that they assess individual farm practices in very different ways [32], thus contributing a diversity of perspectives. Finally consumers and citizens often tend to be excluded or overlooked in innovation activities relating to dairy technologies [20,33].

Consumption patterns—what consumers choose to eat and influences on such choices—are critical factors shaping how food and land use systems evolve. Moreover, public opinion can become a major driver for industry changes [34]; even if attitudes do not influence purchasing behavior (consumer perspective), moral discomfort can be expressed as support for changes in legislation (citizen perspective) [32]. Given the historical fall-out of failing to gauge public risk perception on agricultural issues in the past (e.g., BSE, GMOs) [4], there is a recognition that socio-cultural perspectives, including consumer-citizen perspectives, are inadequately reflected in discussions on more recent on-farm technologies [22,33,35]. Furthermore, it is evident that increasingly urbanized consumer-citizens are asking more questions about production systems, that there is a shift in agriculture from being an activity that is almost wholly rural to one that is more subject to urban influences and that non-farming citizens may have dissonant images of livestock farming, varying from what is perceived as the highly idyllic to the shocking [4]. Livestock products are of particular interest to consumer-citizens in relation to livestock treatments and animal welfare [11,36], as well as other factors including the environmental impact of production, food safety concerns and the social implications of various production methods [36]. Studies that seek to identify consumer-citizens’ perspectives on an “ideal farm” demonstrate that consumer-citizens’ perspectives can be multi-faceted. For instance, in a US study that focused on identifying the ideal pig farm from a consumer-citizen perspective, respondents were reported to consider social, environmental and economic aspects, and to do so from different perspectives. For example, while they considered animal care, with a focus on quality of life, they also considered profitability, emphasizing that the ideal farm should be a profitable business operation and included considerations of workers’ rights and welfare [37]. Another US study that specifically focused on dairy production also found that research participants referred to social, economic and environmental aspects, albeit with a strong emphasis on the animal, both in terms of natural living and animal health, based on ethical arguments and instrumental arguments relating to the consequences of animal care on the quality of milk (and by inference human health) [38]. As noted by the Food and Land Use Coalition “Empowering consumers to make better-informed decisions that are healthier for them and for the planet ignites the whole reform agenda” [15]. A prerequisite for approaches to food systems transformation that empower consumer-citizens is to understand their perspectives.

While, with some key exceptions, there has been minimal activity engaging the general public in conversations around new (digital) technologies in farming, more recent studies have sought to involve farmers as end-users to a greater extent [39,40,41]. This paper focuses on farmers as their decisions and actions have significant impacts on how food is produced, and the quantity and nature of inputs that are available for the next stage in the supply chain. However, they are also significantly impacted by changes in the food system. They currently face increasing risks and pressures as a result of, amongst other factors, climate change, changing public policies and support regimes and increasingly stringent customer demands. Furthermore, demands from the public, through civil society organizations for example, feed into how they conduct their business. As the food system changes, farmers and their employees need to be able to remain in the game, and to be paid fairly to produce healthy and environmentally friendly food, as well as other goods and services. In this way, it is important to consider their perspectives to ensure a just transition.

In addition, farmers have a role as sources of knowledge and as innovators in their own right. The multi-actor approach that is championed by the European Commission’s DG Research, and innovation systems thinking, recognizes that farmers are not only passive recipients of knowledge but that they can/should be actively involved in the co-production of innovations through interactions with researchers, input suppliers, consumers and others [42]. Within co-innovation processes, farmers can be involved in jointly identifying problems and co-creating solutions. Thus, the role and identity of the dairy farmer stands to change considerably with the introduction of new technologies; and yet it will not change at all if there is a resistance to the technologies being developed; farmers are a key target for engagement on agricultural technologies.

In understanding how technologies are designed for and adopted on farms, it is useful to consider farmer agency and farmer subjectivities. Studies of farmers’ subjective perceptions of the ‘good farmer’ have been utilized across a growing range of international contexts in order to identify farmers’ cultural scripts, symbolic capital and social norms [43]. For instance, normative notions of the ‘good’ farmer’, as perceived by farmers themselves, can strongly incorporate productivist ideals within farmer identity [44]. Research amongst Brazilian dairy farmers [38] found that profitability, productivity and efficiency were important components of an ideal dairy farm, however quality of life for farmers and workers was also important for this group. It is important to note that farmers’ subjective perceptions of ‘good farming’ does not necessarily align with other stakeholders’ (researchers, policy-makers and consumers) understandings of ‘good farming’. A number of studies have used the lens of the good farmer to investigate farmers’ decision-making processes and engagement with technology adoption related to animal health on dairy farms [45,46,47], compact calving [48] and environmental management [49]. Other studies have used the concept to understand farmer decision-making processes in switching to dairy farming, including key actors in sourcing information [50,51] as well as to understand the values associated with good farm employment [52]. Other studies, such as [45] identified that farmers’ knowledges and values influence their decision-making process.

## 3. Materials and Methods

The paper is particularly concerned with identifying published academic literature that reports the results of primary research that has been conducted to elicit farmer and consumer-citizen perspectives on dairy farm technologies. (The terms consumer-citizen will be used throughout the paper to refer to consumers, citizens and/or the public unless a more precise term is required and appropriate. Where researchers specifically mention that their research is related to consumers/citizens/public such terms are used.) Relevant literature was identified based on search terms that included adopt*/accept*/attitud*, dairy*, farmer*/consumer*/citizen*, technolog*/innovation*. A preliminary search was conducted in August/September 2021 to provide a framework for the paper. A comprehensive search was conducted in November 2022 to ensure the most up-to-date, relevant published papers were captured. The search was conducted using the library facilities available at University College Dublin, which provides access to wide-ranging databases including Web of Science, ScienceDirect, Taylor Francis Journals, Wiley Online Library, Proquest Science and Technology Databases, CAB Direct and JStor, thus providing access to relevant papers across a wide range of disciplines. Following an initial search, it was decided that for search strings related to consumers/citizens, “farm*” should be included, otherwise literature relating to consumption of dairy alternatives was predominantly identified. Consumer perspectives related to consumption of dairy products or alternative dairy products was not considered to be within scope. Searches were not conducted on specific technologies that have been developed as the authors are not aware of where an exhaustive list of such technologies is available. No time constraint was put on the search. Search strings used include the following:

farmer* AND adopt* AND dairy* AND technolog* OR innovation*
consumer* AND adopt* AND dairy* AND technolog* OR innovation*-
farmer* AND accept* AND dairy* AND technolog* OR innovation*
consumer* AND accept* AND dairy* AND farm* AND technolog* OR innovation*
citizen* AND accept* AND dairy* AND farm* AND technolog* OR innovation*
farmer* AND attitud* AND dairy* AND technolog* OR innovation*
consumer* AND attitud* AND dairy* AND farm* technolog* OR innovation
citizen* AND attitud* AND dairy* AND farm* AND technolog* OR innovation*

Papers that predominantly focused on identifying factors that influence adoption of technologies, measuring the impact of adoption e.g., on productivity or income, all farmers/livestock farmers, elicited the views of experts or stakeholders other than farmers or consumer-citizens, related to technologies such as mobile phone, tablets and computers, related to general thematic areas such as climate change or animal welfare, or were focused on communicating information about production practices were not given detailed attention.

## 4. Dairy Farming Technologies through the Lens of the Farmer and the Consumer-Citizen

In this section, technologies associated with three key stages of dairy production—breeding, feeding and milking are described, and insights from the literature relating to consumer-citizen and producer perspectives are presented. Previous research has identified that consumer-citizens are concerned with farming practices that they perceive influence affective states of animals, the way that animals are treated and naturalness [21]. A range of reasons is provided for such concerns, including a perceived relationship between such practices and milk quality, the environment, as well as animal welfare. It is felt that these three stages of production, in addition to covering much of a cow’s lifecycle, provide an opportunity to assess a range of technologies that could evoke such concerns. Table 1, Table 2 and Table 3 provide details on papers that were identified as having undertaken primary research relating to farmers and/or consumer-citizens’ perspectives on dairy farming technologies at these stages of production. This literature is complemented with additional relevant literature that explains the technology or provides insights on farmer or consumer-citizen perspectives where appropriate. Thus, there is diversity in the nature and variety of literature types contained in each of the three sections, due to different areas of focus in the available literature.

### 4.1. Breeding

For decades animal breeding has largely focused on production efficiency, and it is claimed that animal breeding programs have been responsible for approximately half the observed changes in animal performance [53]. This indicates a high level of industry and farmer adoption and use of technologies produced by breeding programs. A range of reproductive technologies are currently used in dairy production systems, e.g., artificial insemination, embryo transfer, and sexed semen, and a range of hormone treatments are available to aid fertility, or to synchronise oestrus and/or ovulation, and thus calving. These have accompanied the spread of an intensive model of animal husbandry and face different levels of adoption amongst farmers, and knowledge and acceptance is variable amongst consumer-citizens. Ref. [54] hypothesised that it is likely that few consumers are aware of such reproductive management practices, and even if they were aware that they would likely perceive this type of assisted reproductive techniques to be unnatural and unwelcome.

Research on farmers in Australia, New Zealand and Spain found that farmers’ attitudes towards breeding tools is a multidimensional concept [55]. Research on Australian dairy farmers found that they view the utility of breeding tools such as Australian Profit Ranking (APR) and Estimated Breeding Value (EBV) quite positively, albeit with some farmers noting that some important traits do not have EBVs and that APR does not weight traits according to their specific needs [56]. In exploring Danish farmers’ attitudes towards a breeding technology that is not yet implemented (referred to as OPU-IVP-GS technology, combining Ovum Pick Up (OPU) with in-vitro production of embryos (IVP) and genomic selection), ref. [57] found that 76% of their sample (which contained an approximately equal number of organic and conventional dairy farmers) would be likely to use the technology. Most of the farmers saw the technology as beneficial, however, about 1 in 5 has some ethical reservations. In relation to the latter, increasing the speed of breeding refinement to an unnatural level (15% of the respondents), a belief that fertilization should take place without human interference (18%) and a belief that it could “create monsters” (18%) were reasons given. In keeping with the concept of the “good farmer”, 66% agreed that it is important to keep up with new breeding technologies. It is noteworthy that these farmers considered consumer perspectives as well as farmer perspectives, with 44% fearing that consumers could find the technology ethically problematic and lose trust in dairy farmers/farming. However, a Canadian study of dairy farmers, which found that more farmers agreed with the statement that “routine use of [breeding] synchronisation programs is acceptable to me” than the statement “routine use of synchronisation programs is acceptable to consumers”, led the researchers to conclude that while farmers may have an awareness of a lack of public acceptance for certain technologies, public perception does not have a high influence on farmers’ decisions about reproduction management [58].

Sexed semen is proposed as a solution to the production of male calves from the dairy herd that are of limited economic value and are associated with low levels of animal welfare [59]. The term “bobby calf” is used for these calves and it is a focus of many animal rights campaigns. Research in Brazil found that while citizens have a low level of awareness of the practice of culling of newborn male calves, when told about it they reject it outright [38]. It is also of interest to stakeholders within the sector due to reputational risks. Adoption of sexed semen could mean that 90% of the calves born in a herd are female [Holden and Butler, 2018, cited in [59]], thus significantly reducing the production of low-value male calves. However, while some citizens actively seek a solution to bobby calves, consumer-citizens are generally not in favor of technologies that are seen to interfere with nature. A survey of German consumers found that most people have a negative perception of advanced reproductive technologies including sexed semen (the majority of consumers disapproved of the following reproductive technologies; sexed semen (53%), embryo transfer (58%), cloning (80%) and hormone treatment to increase fertility (65%)) [54]. Furthermore, although the technology has been available for a number of years, in many countries adoption rates by farmers have been low due to issues such as low conception rates, low availability and high cost [60]. Drawing on insights from research conducted with consumers in Brazil, ref. [32] point out that sexed semen is a high cost technology, making it less accessible to small-scale farmers, availbale online: https://www.foodandlandusecoalition.org/wp-content/uploads/2019/09/FOLU-GrowingBetter-GlobalReport.pdf (accessed on 26 January 2022).

An interesting aspect of reproduction technologies is that use of one of these technologies can enhance adoption of another technology. Given that the issue of surplus calves on dairy farms is exacerbated in countries where dairying is based on seasonal pasture-based systems due to the emphasis of compact calving, sexed semen may be linked to hormone use. This is because achieving a high degree of compact calving is a complex task, sometimes requiring hormone treatments to achieve it, which may be viewed negatively by consumers. The explosive growth in organic milk in the US in the 1990s is attributed to consumer concerns about hormones [64], and other research in the US reports that consumers prefer natural practices, with the minimum use of hormones [2]. Interestingly a study by ref. [48] demonstrated that compact calving was aligned to the ideal of a ‘good farmer’ in a seasonal pasture-based system, with a high rate of compact calving reflecting positively on the farm and the farmer.

Gene editing is a further development in breeding that facilities faster genetic advancement than the more traditional genetic selection techniques. Gene editing is proposed as a means to improve animal health, animal welfare, and production efficiency and to create the potential to produce milk with reduced allergenic potential, through the controlled manipulation of DNA in a single generation [32,65]. In research on Brazilian consumers, it was identified that the acceptability of gene editing in cattle was increased by perceptions of benefits to animals, and influenced by the perceived distribution of benefits [32]. Some participants in this consumer study in Brazil stated that the premise for accepting gene editing was that the animals should benefit. Interestingly some participants identified economic risks for producers, with benefits accruing to large corporations rather than small scale farmers, while others felt that farmers could take other approaches to achieving the same goal, and in adopting gene editing that they could be in dereliction of their duty to animals. The researchers quote one participant as saying “They don’t want to plant a tree, they want to modify the cattle [for heat resistance] so that they can leave them in the heat, really. They do not want to plant a tree, so they make them [cattle] put up with the sun” (p. 7 of 20). While consumer rejection of practices that are deemed “unnatural” is a consistent theme with the introduction of new food technologies [66], some researchers conclude that gene editing may contribute to the growing public perception of a loss of naturalness in the system, rather than reflecting concerns regarding the naturalness of the technology per se [32]. In this way they argue that concerns about intensification of animal production systems may reinforce these problems, and be the “final straw in solidifying their negative views of animal agriculture” (p. 13 of 20). In contrast to this, the researchers conclude that some participants acknowledge the potential of the technology to tackle animal production, health and welfare challenges. They also conclude that there is evidence to suggest that consumer acceptance and willingness to pay for food produced using gene-editing technology specifically tends to increase when socially beneficial attributes are evident rather than when benefits that accrue solely to the producer [67]. Nonetheless, stakeholders should be aware that acceptance of the technology could be due to resignation rather than trust in the potential of the technology. It has been reported that some participants welcomed gene editing because it was preferable to the status quo [32], while research across consumers and low-input and organic supply chain members, including farmers, across four European countries “confirms that no interest exists within these sectors for innovations based on biotechnology” (p. 1166). In relation to traditional, genetic and genomic selection, research in Australia, New Zealand and Spain found that farmers’ attitudes towards these had two components: traditional selection on the one hand, and genetic and genomic selection combined on other hand [55]. They concluded that farmers’ positive attitudes towards traditional selection does not imply a negative attitude toward genetic/genomic selection and vice-versa and that farmers do not differentiate between genetic and genomic selection despite being quite different breeding approaches with their specific uses, strengths and limitations [55].

Researchers investigated Brazilian farmers’ attitudes to the use of automated behaviour recording and analysis systems (ABRS; i.e., sensor technologies) that could aid oestrus detection, as timely and accurate detection of oestrus is very important for dairy farmers [63]. They found that farmers face practical difficulties in adopting such technologies (e.g., low quality of internet services (33%)), and had concerns about costs. They also found that the farmers were interested in the technology to improve reproductive rates (25%) and monitor production efficiency (25%). Quality of life factors were also important with the technology associated with enabling them to reduce the number of animals to be checked, and to do so more easily. This paper concluded that “farmers believe that ABRS is improving the farm’s routine and quality of their lives as well as reproductive rates” [63] (p. 273). Research on Australian dairy farmers’ perspectives on precision technologies indicates that automatic oestrus detection systems are one of the technologies with the highest levels of expected adoption within 10 years (80% of farmers expected increased adoption rates) [68].

### 4.2. Feeding

#### 4.2.1. Dairy Cow Feeding

Breeding and improved management has resulted in increased yields from cows. However, the increased energetic and nutritional requirements of cows has also resulted in a shift towards more nutrient dense diets, including increases in the amount of grains, pulses and maize in their diets [69]. Thus in many countries, indoor feeding systems such as zero-grazing are increasing while simultaneously the number of grazing dairy cows is declining [70,71]. Grazing is, however, inherently close to the natural behavior of dairy cows [34] and is viewed favorably by consumer-citizens whose concept of an ideal farm is one that incorporates natural living where animals are provided with access to space and pasture [38], and are less stressed. In describing an ideal dairy farm, Brazilian citizens state that such a farm has “*adequate pasture and feeding, as natural as possible*” (Citizen 78);* “… with extensive rearing, natural pasture, so that animals are not stressed*” (Citizen 115) [21]. Avoiding the practice of tethering cows is also viewed positively by consumers [64]. An online engagement study of US and Canadian consumers found that they valued pasture access for cows not only for its benefits in terms of providing grass as a feed to cows; they also cited benefits such as exposure to fresh air, ability to move freely, ability to live in social groups, improved health, and healthier milk products [72]. Interestingly, this study reported that consumers recognize the importance of adequate protection for animals from adverse climatic conditions and identified a significant cohort of consumers who are neutral on their attitudes to pasture access (approx. 17% in this sample). The neutral cohort identified disadvantages as well as advantages of pasture grazing, with a concern that cows could have poorer health, producers would be less economically efficient, additional land would be required and grazing may not be environmentally beneficial. Of the small number who were not in favor of pasture grazing (3.1%), there was a feeling that confinement systems were good for cows because all their needs were met: “*If cows are happy indoors, there is no need to provide pasture*”. The authors concluded overall that participants view access to pasture as desirable but that they recognize that it may be difficult to achieve on some farms for reasons such as a lack of available land, inappropriate environmental conditions for grazing, and concerns about reduced milk production. Indeed, they reported that consumers showed a willingness to combine a mixture of indoor housing and pasture to accommodate the challenge of implementing grazing-based systems on all farms. Similarly, other research found that German consumers accept indoor housing if access to pasture is also provided [73]. Dutch citizens additionally value the image of dairy cows grazing on pasture for aesthetic and cultural reasons [74]. Outdoor systems with grazing animals are perceived not only as more animal welfare friendly by the public but also as more environmentally friendly and more traditional than housed systems [75]. A quote from a participant in the US/Canadian study illustrates this view: “*Rotational grazing gives high production and the cheapest, most environmentally friendly sunlight harvest and fertilizing/waste removal/treatment*” [72]. The extent of consumer-citizens knowledge of pasture-based production is however low in some areas, such that some consumers mistakenly believe that all cattle are pasture-fed and confuse such systems with organic and other production systems [76]. Their awareness of alternatives to pasture based systems is questionable also; only 31% of respondents to a consumer survey in Brazil were aware of the practice of zero-grazing [77]. Regardless of the low level of awareness of zero-grazing however, respondents were able to assess the practice based on factors relating to naturalness, animal welfare, milk quality, environmental impacts and perceived preeminence of productivity and profit over other food animal production goals. Examples of reasons given by these respondents for favoring pasture-based systems include “…*she should be in touch with nature, she is a farm animal*” (R166); *Removing something that is natural for an animal is always a step backward in environmental terms*” (R26); “*With dairy cows grazing natural pasture, the milk is healthier and also the cows are more comfortable*” (R34).

From a production perspective, well-managed pasture-based systems are also generally considered to be environmentally and economically sustainable [78], with many experts valuing pasture grazing “as the most natural, species-appropriate way to keep cattle as it is beneficial both for animal and human health as well as for the environment” [71] (p. 2). Brazilian dairy farmers noted many benefits to pasture-based systems, associating them with high standards of animal welfare, low costs of production, reduced workload for the farmer and environmental benefits. Quotes from farmers undertaken in this research [21] include the following “*Shade, water and pasture during the day and night. This is the only way the animals can be free to express their natural behavior*” (Farmer 127)*; I imagine an ideal farm producing milk with low costs of production, which means, pasture based…*” (Farmer 93); *“…an ideal dairy farm has pasture based production, because it has less workload…*” (Farmer 92). Indeed, there is evidence that cows are highly motivated to access pasture and as a result several Nordic European countries have implemented regulations that require farms to provide dairy cows with access to pasture for specified periods of time, and organic standards in many parts of the world also regulate access to pasture, at least for part of the year [79]. However, research has demonstrated negative as well as positive impacts of dairy grazing systems on the environment when compared with intensive indoor systems; for example, soil can be damaged through treading by cattle in grazing systems [80]. This follows through to more nuanced perspectives amongst different farmer groups. For example, Danish research found that farmers have different views on the impact of grazing on milk yield, with non-grazing farmers associating it with lower yields and seeing this as an obstacle to increased grazing, despite a poor relationship between milk yield per cow and profit [Kristensen et al., 2010, cited in [70]]. Qualitative research on farmers in Western Canada shows the impact of farm specific aspects on farmers’ attitudes towards outdoor access (as opposed to grazing per se) as well as their personal beliefs and values [81]. They found that reasons not to provide outdoor access related to five main thematic areas: (1) adverse climate conditions; (2) concerns about cow welfare; (3) concerns about profitability; (4) lack of suitable farm infrastructure; and (5) ease of management with indoor systems. Ironically the reasons given for providing outdoor access directly mirrored these and were (1) conducive local climate; (2) improved cow welfare; (3) improved profitability; (4) suitable farm infrastructure; and (5) ease of management with outdoor access. Different climatic conductions in different Canadian states and different levels of on-farm investment and associated infrastructure were proposed as reasons for different views on the same practice. Qualitative research of farmers who practiced either intensive indoor feeding (IF) or full-time grazing (FG) found that both groups were equally concerned with animal welfare however they saw high animal welfare being provided through different routes: the IF group was mainly concerned with adequately fulfilling animal requirements through feeding concentrates (high-yielding Holstein dairy cows can show energy deficiencies unless their diets are supplemented to a large extent with concentrates [82]) while the FG group was focused on the positive aspects of grazing on animal welfare [Baur et al., 2010, cited in [70]]. Overall, there is a recognition that there are constraints to providing pasture, particularly on farms that do not have an adequate land base or on farms whose land base is vulnerable to impacts of grazing cows. Ref. [72] concluded that producers in their US/Canada study appreciate the benefits of providing access to pasture and that they supported it in principle, but that they felt limited in their ability to do so in practice.

Differences in perspectives on pasture feeding between farmers and consumer-citizens was highlighted in a review [70]. While they reported that milk with a pasture-fed label resulted in a higher consumer price than milk without a pasture-based label, they reported that the establishment of the German label was difficult because the dairy farmers were concerned that the program would lead to discrimination against all-year housing. Other Willingness to Pay (WTP) studies have reported that such market differentiation can result in a lower WTP for conventional products rather than a premium for differentiated products. Thus, adoption of some innovations that are seen as beneficial by consumers can have a positive impact on some farmer groups but simultaneously they can have a negative impact on other farmer groups.

All systems of production are coming under increased scrutiny regarding greenhouse gas emissions and more notably the production of methane from cows is to the forefront. Dietary characteristics and fermentation conditions in the rumen are factors identified as influencing methane production [83]. Dietary measures investigated to reduce the methane loss from dairy herds involve the manipulation of the cows diet through the inclusion of additives (e.g., ionophores, organic acids, and plant secondary metabolites (e.g., condensed tannins), oils, red seaweed) as well as improving forage quality and increasing concentrates fed [83,84]. Ref. [84] stated that the potential use of plant extracts including seaweed are seen as a natural alternative to chemical additives and are well perceived by consumers, however the study did not investigate consumer attitudes towards dietary manipulation. Overall, there appears to be limited research focusing on consumer-citizen perceptions regarding methane reduction strategies of ruminants, with an expectation that they will accept such technologies because the end result is desirable.

This seems to be mirrored in relation to farmer perspectives. While research has been conducted on farmers’ knowledge and attitudes towards climate change, and some research has been conducted on farmers’ preferred mitigation options in the Netherlands, including dietary manipulations involving by-products and increased maize feeding [88], their attitudes on dietary additives for the purpose of reducing GHGs do not seem to have been investigated. Ref. [88] concluded that farmers tend to choose mitigation options that are relatively simple and either cost effective or have only relatively small additional costs. This could be the case for feed additives that reduce GHGs but other factors could also be involved. Ref. [84] stated that farmers will adopt a practice (such as methane reduction strategies) only if there is a positive economic impact on animal production and farm profitability, but there is evidence to suggest that this is not always the case. Investigations of how farmers’ engagement with conservation tillage is shaped by their identity as a good farmer found that while economic capital played an important role in farmers’ decisions to adopt conservation tillage, cultural and social capital were inextricably linked to its development [89]. Furthermore, the concept of the good farmer embodies the good cow with many studies showing that farmers have an emotional attachment and pride in their cows [90]. Research investigating Swedish farmers’ perspective of antibiotic use found that the farmers interviewed had a sense of responsibility for their cows, which was central to their farm management, and is an important factor determining agricultural practice [46]. These factors could also be at play in relation to methane abatement strategies. However, there seems to be a knowledge gap in this area.

#### 4.2.2. Dairy Calf Feeding

Separation of the cow and calf immediately or shortly after birth is routinely practiced on dairy farms [9,34]. It is however the focus of increased interest among the public because of calf welfare concerns, with high profile campaigns by animal welfare groups targeting this practice. Consumer-citizens who object to the practice believe that early contact is important for both the cow and calf, that it is natural and that separation is unethical. Brazilian consumers made the following statements in respect of the practice “*Contact with the mother is essential for all species*” (R398); “*It is not right to separate cows from their young just to increase milk production*” (R216) “*I believe that the stimulus generated by the calf suckling and the emotional connection between them are beneficial for both the quality of the product and the health of the animals*” (R383) [77]. Similar sentiments were expressed by the public in research conducted in North-America/Canada with a view amongst supporters stating that the industry can and should accommodate cow-calf pairs [9]. Despite this interest, researchers found that urban Brazilians were generally unaware of this practice (33% of their sample was unaware of the practice) [77], and a study in North America/Canada found a lack of consensus regarding acceptance of this practice; they found 44%, 48% and 9% of their respondents chose “yes”, “no” and “neutral” respectively to the question “Should dairy calves be separated from the cow within the first few hours after birth?” [9]. Research in the US and Germany [13] also found a lack of consensus on the topic; they identified three segments labelled “Late”, “Unsure” and “Early” referring to the preferred time of separation, and found consumers from both countries in each cluster. This could be explained by Dutch research; these authors highlight a tension felt by consumers between modernity and naturality in relation to the separation of the calf and cow and calves being fed with milk replacer rather than their mothers’ milk. They cite a quote from a Dutch consumer from earlier work [4] “*Production comes first. I understand that a farm has to function like a business and that the milk production needs to be as high as possible. But I feel a bit of resentment too. Because what is best for the animals? As humans, where are we going*?’ Research in the US and Germany found national differences in preferences; they found German consumers were generally more in favour of later separation (69% of the sample) compared to US consumers (55% of the sample) based on initial questioning [13]. The researchers speculated that these differences may be a consequence of differences in values, which are influenced by cultural norms within societies. Interestingly they also found that when consumers in both countries were presented with 22 different arguments for and against early and late separation, there was a decline in the numbers favouring early separation, an increase in the unsure responses and a decline in the responses at the extreme end of the scale for later separation. Consumer cited responses in favour of early cow-calf separation include welfare benefits in research undertaken in the US and Canada “*I think farmers should separate the calves from the mothers not only for the calves’ safety but so the mother can rest*” (P930-CR) [17].

Research has reported that consumers see farmers as being at the center of such dilemmas, and as having a responsibility to “handle these dilemmas, resolve the conflicts and maintain a desired balance between modernity, tradition and naturality” [4] (p. 1461). There has been increased interest in extended cow-calf contact by the farming population as well as researchers and advisors, but while some European farmers provide extended cow-calf contact [34] dairy farmers were found not to be favorably disposed to it in research conducted in six European countries [85] and Brazilian farmers have concerns regarding animal welfare, labor, and the suitability of the dairy system for providing extended cow-calf contact [77]. Farmers in New Zealand had similar concerns relating to (1) poor animal welfare for both cow and calf (relating to mastitis in the cow, inadequate colostrum for the calves, lack of shelter for calves while outdoors with cows and increased stress to both due to delayed separation); (2) increased labor and stress on staff; and (3) requirement for system level changes, relating to infrastructure and herd management [34]. Some illustrative quotes from this research include: “*The reason I don’t do it* [keep cows and calves together] *is the failure rate of calves not feeding off their mothers. And the importance of them getting the colostrum within the first few hours*” (C-53); *If the cows and calves are together and you get bad weather, there is no shelter for them. When you take them off, at least they are getting fed twice a day*” (C-16); *Once they get that bond, it is hard to keep the cows in* [the paddock]*, I think the cow is under stress for longer, and she bellows all day and night*” (C-15); *If it is going to take twice as long as what you are already going, then there has got to be some pretty decent benefits*” 9C-44; *“We are all on time restrictions and I think often what could be better for the animal could be quite detrimental to the farmer by way of mental health and added pressure*” (C-33); “*Under health and safety you couldn’t do it* [keep cows and calves together]. *I would knowingly put my staff in harm*…[if I did that]…*Cows get more protective. You couldn’t do it with a clear conscience* (C-7). Similar to pasture-based production systems, while some of these farmers were theoretically in favor of extended cow-calf contact, they cite practical barriers to implementation. Condensed, seasonal calving patterns, associated with pasture-based dairy systems, are the root of many of these concerns. Echoing issues raised with AMS systems below, farmers note that changing the cow-calf system would require substantial system change rather than merely changes to the calf-rearing process “*I would have to change the yard management, grazing management, insemination times, bull management, and also the surviving of calves will be a little bit less.*” (C-41). A fundamental objection however to prolonging cow-calf contact related to farmers’ fundamental views of their role as farmers; many see their role as relating to milk production as opposed to rearing calves. “*We are about producing milk. That is why we wouldn’t leave them on for longer*” (C-17). Interestingly, in contrast to this, lack of confidence in the mothering ability of the cow was another reason for early calf removal, with some farmers citing breeding as a cause of this; “*I’m not saying that it has been done deliberately, but if you look at the mothering ability of cows today compared to the cows that were bred 30 years ago, you would find the mothering ability has been quietly eroded*” (C-27). These farmers also recognized that different systems may be appropriate in different contexts and see the wider context of dairy farming “*It is more area-specific the way you can do things. What we can do on the west coast* [is different from what farmers in other areas can do]…*It can even be farm-specific, not always area-specific*” (C-25). Many of these concerns were refuted by a small number of farmers who practice cow-calf contact, notwithstanding agreement about the need for additional infrastructure and changes within the wider system for successful adoption.

The high labor requirement for calf rearing, particularly on seasonal calving farms, has resulted in increased interest in using technology and automation to reduce the labor requirement associated with calf rearing. Automatic calf feeding systems are increasing in popularity primarily due to claimed economic gains arising from labor savings [91]. Increased feeding frequency, gradual weaning and socialization benefits for the calf [92] means that automated feeding can come closer to mimicking the way calves feed and behave in nature, potentially increasing animal welfare. Although there is the potential for increased illness with automatic calf feeding systems if not managed correctly (e.g., due to increased production of urine and faeces), farmers in England have identified one of the benefits of the use of automated milk feeders as alerting them early on to signs of calf illness—such as slow drinking or lower feed consumption [86], however the research did not specifically address this technology. Nonetheless, the research provided additional insights on their perspectives regarding calf housing systems. It found that farmers used a variety of group sizes (including individual housing early on) when housing calves, with variation largely dependent on the space available to rear calves and the labor-intensiveness of different systems. Individual hutches were considered particularly demanding, but worth the extra labor for improved calf health. Moreover, insights can be gained from other research. The standards of ‘good farming’ are embodied in livestock or the ‘good cow’ [90]. Therefore, essential to achieving the ‘good cow’ is ensuring excellent care and management of calves from birth through good stockmanship. In addition to the traditional stockmanship skills required to rear calves, the adoption of automatic calf feeders will require the development of new skills and knowledge by the farmer. It will require the farmer to be competent in data collection and interpretation to compensate for the reduced direct contact with calves in order to produce a ‘good calf’. In a study investigating producers’ perspectives on neonatal care of calves, farmers were intrinsically motivated to provide good calf care by their sense of pride and morality, by social obligation to other dairy-industry stakeholders, and sometimes by the economic benefits for their herd [93]. Although ref. [94] concluded that participants in their study did value their calves, they noted that some producers did not highly prioritize calf health and productivity outcomes. This could be due to a lack of resources including inadequate facilities, a lack of clarity about calf care as well the time and effort necessary to care for newborn calves [93]. Therefore, when implemented correctly automatic calf feeders could allow farmers to improve overall calf care thereby increasing productivity. However, there is a lack of research regarding the opinions and perspectives of farmers on automatic calf feeders as well as data quantifying the actual uptake of the technology.

While there does not appear to be any research on consumer-citizen perspectives on automatic calf rearing systems, a study in the US found that group housing of calves was preferred, followed by pair housing with individual housing least preferred [87]. Reasons cited related to increased socialization *(“the calves can play and socialize which is important to all animals*” (PY427)) and space allowance. Of the small numbers who preferred individual housing, calf health (“*best for controlling diseases and nutrition intake*” (PA1124)) and having their own private space were provided as reasons. Furthermore, consumer-citizen perspectives can be anticipated from other research. Although much of the public is unaware that cows and calves are separated shortly after birth, when presented with the information, the main objection is its unnaturalness [9,95]. The definition of naturalness is shifting according to some research, and while the older generations of the public may consider technological solutions to be a further deviation from naturalness and a departure from dairy farming’s agrarian roots, the definition of “naturalness” for younger generations may well have expanded to include technology [96]. In that regard, the adoption of automatic calf feeders, which allows for autonomy and some similarity to natural behavior, while simultaneously improving individualized care, could improve public perception of calf rearing systems. In this way, it may serve to counteract a misalignment of the needs of the public with the needs of the farmer [96]. This, however, has not been subject to empirical research.

### 4.3. Milking

The herringbone and rotary parlors are early examples of technological advances designed and led by farmers, which enabled the expansion of dairy farming. This expansion was seen by many as necessary to increase and stabilize food production [97]. The change from hand to machine milking provides a historical context for automatic milking systems (AMS, or robotic milking machines) and the same concerns that arose then are seen to persist today with the introduction of AMS [98]. Dutch research, however, finds that “the entire practice of dairy farming has been reorganized around this device” [99] (p. 3). This sentiment is echoed in research with Norwegian dairy farmers in which some of them see the technology as implying a completely new way of working, requiring them to be more proactive in relation to animal health and hygiene, using the data provided as a management tool, and focusing on both herd averages and individual cows in managing performance [100].

The first commercial AMS emerged in the early 1990’s in the Netherlands and since then they have been adopted at an increasing but variable rate in many countries including across Europe, North America, Australia and New Zealand [99]. Initially AMS was more commonly operated in intensive indoor feeding barn systems where cows have limited access to grass but since 2001, they have been incorporated into pasture-based systems worldwide [101]. These systems were established as fixed milking parlors but mobile milking systems (MMS) are also available, enabling them to be used in free-range or rotational grassland systems [102]. In Europe and the US, the majority of AMS are used on small and mid-size farms (<500 cows) [103,104,105]), possibly to avoid or reduce the need to hire non-family labor and/or to increase productivity without increasing labor input [104]. AMS are, in part, a response to some of the issues associated with conventional milking such as health, welfare and labor [98]. Despite high costs, AMS are increasingly popular amongst farmers for labor-saving and lifestyle benefits, including more time for family and recreation [106,107]. Increased flexibility, as opposed to time gained, was identified as a key benefit of the technology [99], with farmers in Norway reporting flexibility as more important than labor saving [100]. However some farmers found that AMS provided less flexibility than expected, because of the need to be constantly on-call [98,100,107]. Research on Norwegian dairy farmers indicates that while flexibility is the greatest advantage of AMS, allowing farmers to spend more time with family and friends, and to get more sleep, they acknowledge that flexibility can come at a price and that it also comes with responsibility [100]. The researchers quoted a Norwegian farmer as saying “*Farmers who consider investing in a robot often ask me: How much time do you need in the cowshed? I answer: As long as possible…You get more flexibility, but its freedom with responsibility*” (p. 114). Ref. [98] (p. 131) state that claims regarding the benefits of AMS, to both the farmer and the cow, are “certainly contested”. The finding that Australian farmers expect lower levels of adoption of AMS compared to service providers (60% vs. 79% respectively) supports this view [66]. On the positive side, research with Norwegian dairy farmers supports a view that the data provided by AMS makes farming more interesting. A Norwegian farmer is quoted as saying “*Robotic milking has definitely made farming more interesting. You get a lot of information about each cow…it’s really a good management tool*” [100] (p. 113). The researchers also note, however, that farmers are selective about the data used and that some find it difficult to utilize all the data provided. Adoption is also associated with being progressive by these farmers; “*To keep up the interest as a farmer something new much take place on the farm from time to time…you have to develop the farm, you can’t just stand still*” [100] (p. 114). This research also reported on how farmers “domesticated” the technology, addressing challenges relating to being constantly on call, and illustrating the dynamic processes involved in successful innovation.

The milking process of AMS is promoted as a fully automated and voluntary process with no set defined milking times [108]. In this way it is presented as allowing cows to engage in natural behavior. Instead of human handlers herding cows to the milking systems two or three times a day, cows are incentivized to enter the stall of the AMS unit when they want to by the provision of feed on entry. Relative to conventional milking systems, the milking process in AMS is consistent and the milking routines are predictable for the cows, which is a prerequisite for successful milking [109]. The flexible and the voluntary nature of the AMS should be conducive to the societal demands of improving animal welfare. Ref. [99] in reporting on farmers’ interviews in professional magazines, confirm that farmers are convinced that a robot approximates a natural situation. However, there are questions about how AMS influences cow welfare [110], with challenges identified in relation to udder health, lameness and disruption of the natural behavior of cows [109,111,112]. Moreover, while the technology is presented as a voluntary system, designed to give cows freedom and autonomy and enable the expression of more natural behavior by cows, it has been argued that “cows’ bodies, movements and subjectivities are trained and manipulated in accordance with a persistent discourse of agricultural productivism…[…] and technological interventions [specifically robotic milkers] […] contribute to a re-capturing and re-enclosure of bovine life which counters the liberatory discourses which are used to promote robotic milking” [110] (p. 131). Cows are herd animals preferring to perform their activities, such as eating and resting, synchronously but AMS requires that these tasks are performed individually, which could disrupt the natural behavior of cows [109,112]. Ref. [111] highlighted that although the milking process is automated, the role of a competent stockperson has not diminished and is vitally important in ensuring that animal welfare is not compromised. European consumers have previously been found to be concerned that increased robotization on farms could lead to the traditional farmer role being replaced by machines, with less human attention directed towards animals and thus, negative welfare consequences [35]. However, using the Ethical Matrix as a guiding framework, it was found that UK consumers considered AMS largely acceptable, although they were concerned with animal welfare [113].

The development of AMS can be seen as a shift in the concept of good farming from caring for the animals to allowing the animals to take good care of themselves [99,109,116]. This change in cow agency and subjectivity tends to suggest a sort of automation of human activity too [98]. Indeed, it is suggested that both bovine and human agency and subjectivity are entrained and reconfigured with the use of AMS technology [98]. Ref. [110] established that the adoption of AMS technology results in a renegotiation of the established ethical relations on a dairy farm due to the change in the corporeal relationship between cows and humans. The authors suggest that AMS redefines the notion of care in dairy farming, thus changing the understanding of what constitutes a ‘good farmer’ or ‘good stockmanship’. Similarly, researchers suggest that the professional identity of farming is changed from manual labor to an office-based job with the adoption of AMS technology [99]. In conventional milking systems, good stockmanship can be as seen as the knowledge and skills that evolve from prolonged contact with animals during milking whereas with AMS there is less direct contact with animals and instead the farmer is more reliant on technology and data to identify problems [95,109,116]. In that regard, AMS technology creates a shift in identity as farmers are expected to become skilled and knowledgeable in the use of data collection and interpretation while combining it with traditional stockmanship skills to produce a better farming system [98]. A ‘good AMS farmer’ is therefore defined as one that combines the traditional stockmanship skills with data collection and interpretation skills [98,99]. In this way, AMS does not replace traditional stock keeping skills, rather AMS can enhance stock keeping but this is dependent on the extent to which the stock-keeper is able to adapt their behavior through changes in transformed dairying practices [18]. Ref. [18] suggest that where the stock-keeper is able to adapt to the AMS, the hybrid capital of humans, animals and technology can be successfully invested to improve productivity and herd health and welfare, and to enhance human quality of life. With farmers themselves placing increased emphasis on work-life balance, AMS can provide an alternative when labor is unavailable (the absence of a successor or hired workers) or provide a way of continuing to farm after a certain age at which manual labor becomes more of a strain [99].

Research on UK consumer perspectives on AMS found that 60% of the consumers in their sample believed that AMS use would benefit farmers [113]. However, while 25% believed that the technology would benefit cows, 50% expected cow welfare to be reduced. When asked about impacts on safety of milk for consumers, 20% believed it would improve safety of milk for consumers, 43% expected no change and 25% expected it to be reduced. Approximately 10% of consumers gave “don’t know” as a response in relation to each of these issues. Given the wider systemic changes, as well as changes in the farmer-animal interface expected above, there seems to be a gap in relation to understanding nuanced consumer-citizen perspectives on AMS.

## 5. Discussion

Many new technologies and innovations have been introduced to the dairy sector over the years, and more will be required in the future to ensure that dairy production is part of a sustainable food system. It is clear however that successful introduction of such innovations on a widespread basis will not be easy. The problem of technology adoption has for decades been addressed in the social science and extension literature, traditionally with a focus on ‘barriers’ such as knowledge deficits and cultural preferences and, more recently, focused on the importance of recognizing ‘adopters’ as co-designers of technology, with valuable knowledges and end-user insights for the design process. The successful introductions of innovations on a widespread basis is further complicated because sustainability encompasses economic, environmental and social aspects, the simultaneous fulfilment of which can involve trade-offs. Furthermore, more specific to dairy farming, it is accurately explained by [4] that “the (sociocultural) sustainable development of livestock farming is not an objective concept, but [one] that it is socially and culturally constructed by people in specific contexts”. Moreover, how individuals across different actor groups perceive and evaluate technologies is socially constructed and shaped by beliefs and expectations [117]. This, in turn, as is evident in the review presented in this paper, means that different actors are likely to have different perspectives on both problems and proposed solutions, and that these are likely to change over time.

This review has found that a wide range of authors have employed diverse methods to assess consumer-citizen and farmers’ perspectives on a range of dairy technologies. Notwithstanding the fragmented nature of this research, and the limitations of the methods used, it found that in addition to expected differences in perspectives between the two actor groups, there are diverse perspectives within each group, as well as areas of agreement between the groups. Moreover, considering the consumer-citizen perspective, it is clear, as has been reported elsewhere [118] that consumer and citizen perspectives can cause internal tensions within individuals. A potential conflict is illustrated in the case of sexed semen above: as a ‘citizen’ an individual may support the technology due to its potential to mitigate animal welfare issues associated with surplus calves; however, as a ‘consumer’, there can be an aversion to reproductive technologies and a distrust of biotechnology in general. While some researchers [13] expect citizen perceptions to be more diverse than farmer perceptions, it is clear that farmers are not a homogenous group either, and, additionally, they can occupy other roles alongside the role of farmer, including vets, farm advisors, and input suppliers. In a similar way to the cognitive dissonance seen in relation to consumer-citizen perspectives, this can lead to internal conflicts between the farmer as a “professional” or “expert” and as a “practitioner”.

The OECD [31] in the context of developing policies for livestock systems, state “scientific facts, including from independent advisory groups, play an important role but are not always widely accepted by the public or stakeholder”. The deficit model approach which viewed a lack of knowledge as the primary barrier to public acceptance of technologies is now considered an outdated model, replaced by years of sociological and psychological studies demonstrating how different actors’ perceptions of risks and benefits of new technologies are shaped by a myriad of values, experiences and beliefs [119]. Research cited in the preceding sections shows that citizen-consumers assess technologies across a range of dimensions, and that they can consider both positive and negative impacts simultaneously. It is also clear that many consumers can understand the importance of context in considering what is sustainable, with some acceptance of farm-specific solutions. This was particularly evident in the section on dairy cow feeding above. Furthermore, they can have a nuanced understanding of the issues, for example opposing a single-minded focus on animal health at the expense of natural living [120]. Ref. [64] however highlighted that consumers have complex and conflicting motivations when making ethics-related food decisions, and consumers may simultaneously define sustainability in conflicting ways or desire conflicting ideals, such as valuing both technological advances and undisturbed nature/naturalness in an agricultural system [74]. This suggests that consumer-citizen perspectives on individual technologies should be assessed in a wider decision-making context within which consumers engage.

Some similar issues are evident when considering producers’ perspectives with tensions as well as areas of agreement evident with this cohort. Different groups of farmers have different perspectives based on farm and farmer-specific characteristics (e.g., those who feed cows indoors and those who provide access to pastures) and interestingly even where their views on what might be desirable are similar in principle, this may not be consistent with what they do on their own farms for a range of legitimate and practical reasons. This is illustrated in the case of pasture feeding above, with economic factors and land access being significant factors hindering implementation of what might be generally considered a desirable practice.

Fear of industrialization of farming with the introduction of technology and innovation is a recurrent theme in the consumer-citizen literature. The literature cited here suggests that views on what industrialization means in the context of dairy can evolve over time, with some newer technologies being viewed as potentially more “natural” than the technologies that they are replacing. This is illustrated in the section on automatic calf feeding above. However, in general, consumers have been found to be concerned by the concept of robots and machines replacing the traditional role of the farmer. Rather than seeing technology as a data-driven decision tool used by farmers, consumers tend to view the two as being in competition and the expected impact in terms of diminished power/agency of the farmer is a concern [35]. Thus, societal trust in farming technology, and the perceived transformational role it could have for food production systems, remains a key challenge These concerns, along with the link consumer-citizens make between on farm practices, animal welfare, product quality and human health, and to a lesser extent to environmental impacts, indicate the value of including such actors in anticipatory roles in designing and implementing innovations for a sustainable food system.

When considering farmer and consumer-citizen perspectives simultaneously, it is clear that there are some tensions between the groups. However, we also identified areas of agreement. Noting both tensions and areas of agreement is important for designing and implementing strategies, technologies and shared practices that are responsive and inclusive—“understanding broad areas of social consensus, as well as disagreement, will help to identify methods of bringing industry practices better in line with public expectations” [72]. Moreover, both groups similarly experience conflicts within the context of their own value systems and contradictory expectations where the development of smart farming is concerned. This is to be expected considering the experimental nature of technological development and the way in which innovation inevitably challenges existing values, perceptions, beliefs and preferences, etc. As explained by [4], “tensions [exist…] between modernity, traditions and naturality—‘the MTN knot’—each of which has positive and negative faces”. Interestingly, it was reported that Dutch consumers valued the agricultural system in the Netherlands as it combined “apparently contradicting aspects such as technology and nature within one system” [74] (p. 32). However, as noted by ref. [2] and concluded by ref. [4], the general public ‘wants it all’: they prefer naturalness and tradition, but also value modernity in dairy production. While disruption and transformation in agriculture in the past was focused on modernization and industrialization, it is clear from the perspectives reviewed in this paper that technological-driven changes in the dairy sector will also have to embrace “returns to the past”, and give due respect to “naturalness”, principals deemed important by key actors in the value chain.

Overall, the results raise two questions with respect to diverse actor perspectives: (1) can such differences in perspectives be addressed and (2) should such differences be addressed? Answering these questions requires an understanding of the causes of the differences in addition to the identification of the differences. The OECD [31] identified three sources of friction in decision-making processes involving different stakeholders, differences relating to knowledge, interests and expectations, and values and some researchers [13] agree that differences in knowledge and a lack of shared values contribute to differences in opinions between consumer-citizens and those involved in livestock production. Where they arise due to differences in knowledge, some researchers propose that efforts should be put into educating the public so that can they better understand the nature of farm practices and the reasons for their use on farms [54,77]. Concepts such as edu-tainment and edu-tourism have been promoted in this context [121]. This is based on an assumption that most consumers have limited knowledge and experience relating to agriculture, and thus that exposing them to farms can give them a realistic impression about what farming actually entails [74]. This assumption is verified by research that identified a disconnection between consumer expectations towards mountain production systems and reality [122]; when conducting research on how consumers perceive products from traditional mountain dairy farms they found that appreciation of certain husbandry and management choices did not translate into a recognition of what they looked like when images of traditional husbandry systems were provided. However, in relation to resolving concerns about a complex issue such as animal welfare, it has been argued that relying on public education is not likely to be very effective because of the low ratio of “naïve public to industry insiders” [12]. Moreover, as found by research on US and German consumers [13], clusters with different perspectives attend to different types of information and arguments, often selecting information to support initial positions, and reflecting people’s moral intuition. Furthermore, views about such perceptions are formed based on prior experiences and values, as much as knowledge and facts. Thus educational efforts, which often are included in government policies, are unlikely to consistently result in the desired effects, heading off criticisms about animal production practices, when differences in values rather than ignorance is the cause of difference [13,123]. It is interesting to note that educating farmers is generally focused on providing them with technical expertise, with a limited focus on social learning whereby farmers could obtain a better understanding of what is required by the market and society. The concepts of edu-tainment and edu-tourism could thus be considered for their role in educating farmers as well as the public, potentially contributing to further alignment in perspectives. Differences relating to knowledge can also relate to a lack of agreement on what the facts are and what the evidence provided by science means. It also assumes that scientific information is unbiased and that science-based information is the only source the public should use to assess agriculture. Some researchers argue for greater transparency and communication, rather than education per se, as they believe that when consumers are kept unaware of dairy industry practices, the potential for miscommunications and misunderstandings surrounding sustainable dairy grows [124]. They caution that such initiatives need to be well-planned, founded on an understanding of consumer expectations regarding sustainability practices, and supported by evidence; “greenwashing” or other misrepresentations of sustainability efforts can easily lead to consumer backlash. Aligned with the principles set out by RRI [27], any government policy interventions should consider the consequences—positive, negative or unintended—an intervention could have [125]. One manner of doing this is to lever inclusion and participatory exercises, which incorporate consumer-citizens and farmers so as to ensure their diverse values and needs are reflected in policy development.

Where groups have diverging interests, representative groups come to the fore. These can be legitimate advocacy groups (e.g., civil society organisations in the case of animal welfare) or influence-seeking interest groups who pursue their special interests ahead of public interests (e.g., representative bodies that look to expand dairy production without taking account of climate impacts). Diverging interests can be addressed through inclusive and transparent decision-making processes. Unaligned interests can be addressed also through providing some compensation to those who are negatively affected. Where farmers incur extra costs in adopting technologies that can have a negative impact on performance but a positive impact on the environment, e.g., as may be the case of methane reduction strategies, policy instruments can, and arguably should, compensate farmers for subsuming their financial interests in favor of public good.

Addressing differences in values is more challenging. However, where shared values exist, there are significant opportunities to implement practices that are seen as desirable by a range of actors. Shared values amongst companies, governments and social organizations relating to grazing and the benefits of it, resulted in the establishment of the Grazing Agreement in the Netherlands in 2012. This initiative, involving a contract among more than 80 organizations (companies, governments, and social organizations) to promote grazing practices, is credited with 82% of dairy farms in the Netherlands practicing grazing in 2018. In addition to financial incentives for primary producers, research, education and extension are important components of the program. These are also important for government policies, which often focus on financial incentives, and acknowledge that top-down regulatory interventions (such as regulation, incentives or punishment) need to be accompanied by bottom-up interventions that tackle the motivation, values and capability of individuals to engage with new technologies and practices [126]. Where values are not shared, differences can still be reconciled. This can be achieved through creative thinking to find specific actions that can be supported by people with different values. This requires high levels of engagement, and deliberative approaches can help to build societal consensus, or at least to clarify issues. In complex cases, this can involve a series of multi-stakeholder consultative processes [31].

Differences in perspective between and within groups is not in itself problematic. Indeed, “tensions between diverging interests (and hence interest groups) are unavoidable in diverse and pluralist societies, and much of political decision-making involves a search for compromises or grand bargains which can reconcile diverging interests in society” [31]. Indeed, diverse perspectives can result in better solutions through challenging assumptions. However, differences can be problematic, and need to be addressed, if one interest group has a disproportionate influence over decision-making and if certain groups are not included. Moreover, it is important to recognize that the responsibility for developing sustainable food systems is not vested in one actor; rather it is allocated jointly between farmers, policy makers, regulators, different actors in the food chain, consumer-citizens, etc. [11,127]. Thus, it is not a matter of blindly agreeing to do what one group wants or expects; some minimum level of consensus is required to progress. Without this, innovations and technologies that neither fit the farming context nor meet consumer needs could be developed. In addition, it could also result in an “unfunded mandate” whereby producers are required to adopt practices that result in additional costs to them without any extra return. Such an approach is therefore unlikely to be economically or socially sustainable. Arguing for solutions between farmers and non-farmers in agricultural governance, ref. [128] discusses the need to recognize a “shared fate, interdependence, and mutual responsibility”. The inclusion of all perspectives (encompassing knowledge, expectations and values as well as attitudes and behaviors), and as highlighted in this paper specifically, the inclusion of farmer-consumer/citizen perspectives, is essential in decision-making process regarding technology and innovations in the dairy sector, with responsiveness necessitating acknowledging differences while striving to find common ground.

Inclusion however should not be viewed as a quick-fix or a panacea for consumer resistance or farmer non-adoption of different technologies. It should be viewed as a mindset and a commitment to ‘doing’ research and innovation differently. Increasing the inclusiveness of the innovation process will ensure a more fair, balanced and multi-dimensional approach to the development and implementation of technologies and innovations on farm. Given the agricultural production system impacts of some technologies (e.g., automatic milking systems), as well as wider food systems impacts (e.g., in relation to the roles and identities of farmers, product quality), this wider approach will be essential to helping to identify and address unforeseen and unintended consequences. At the same time, inclusion must be carefully managed. Inclusion “is not a prerequisite to success; as well as being time consuming, this may create uncertainty if roles and objectives are not clear from the outset” [22]. Social sciences can support the careful management of this process of engaging and bringing diverse stakeholders such as farmers and consumer-citizens into the inner circle of innovation and research. Moreover, due to their expertise in the methodologies that support co-design processes, social sciences have a key role to play in facilitating inclusive innovation to support responsiveness in the dairy sector [127]. Overall, while agreeing with calls [4] for ongoing collaboration between social and animal scientists in order to develop livestock farming systems that are more socio-culturally sustainable, we call for a concerted effort to ensure anticipation, inclusion, reflexivity and responsiveness in developing innovations for the dairy sector of the future.

## 6. Conclusions

Innovation has always been a risky process. As society places greater demands on dairy production systems to produce a range of goods and services beyond basic food products, and as researchers and their funding bodies recognize the need to embed principles of responsible research and innovation, identifying, understanding and taking account of (to some extent at least) the perspectives of diverse stakeholders will become increasingly important. As this review highlighted, these stakeholders have both similar and divergent perspectives, so this will not be an easy process. Moreover, while consumer-citizens can be easily involved as research subjects, and vocal civil society organizations exist to engage on some issues (e.g., animal welfare), unlike farmers who have representative bodies, the route through which consumer-citizens can be actively involved is not so clear. Deliberate and concerted efforts on the part of policy makers, research funding bodies, dairy sector organizations or others is required.

## Figures and Tables

**Table 1:** Overview of primary research on consumer-citizen and farmer perspectives on selected on-farm dairy technologies (general and breeding related).

Citation	Country	Respondents	Method	Technological Focus
Boogaard et al., 2011 [4]	The Netherlands	Public	Online survey	Dairy farming practices generally
Dalcq et al., 2020 [61]	Southern Belgium	Dairy farmers	Online survey	Ideal future farm
BREEDING RELATED				
Cardoso et al., 2017 [38]	Brazil	Urban citizens	In-depth interviews	Culling of new-born male calves
Denis-Robauchaud et al., 2015 [58]	Canada	Dairy farmers	Survey: online, email with link, hard copy postal	Reproductive technologies
Lund et al., 2021 [57]	Denmark	Dairy farmers	Online survey, with postal/telephone options available	Reproductive technologies
Martin Collado et al., 2015 [56]	Australia	Dairy farmers	Online survey	Genetic selection
Martin Collado et al., 2021 [55]	Australia, New Zealand, Spain	Dairy farmers (and beef and sheep)	Face-to-face and online survey	Genetic selection
Nicholas et al., 2014 [62]	Belgium, Italy, Finland, United Kingdom (UK)	Consumers, farmers and supply chain members	Group interviews	Genetic modification and transgenic organisms
Pieper et al., 2016 [54]	Germany	Consumers	Face-to-face interviews	Reproductive technologies
Viera et al., 2021 [63]	Brazil	Dairy farmers	Semi-structured interview, phone/online questionnaire	Automated behaviour recording and analysis (oestrus alert)
Yunes et al., 2021 [32]	Brazil	Citizens	Face-to-face/online survey, in-depth interviews	Gene editing

**Table 2:** Overview of primary research on consumer-citizen and farmer perspectives on selected on-farm dairy technologies (feeding related).

Citation	Country	Respondents	Method	Technological Focus
Busch et al., 2017 [13]	America, Germany	Public	Online survey	Cow-calf separation
Cardoso et al., 2019 [21]	US	Public	Online questionnaire	Housing/pasture access
Cardoso et al., 2017 [38]	Brazil	Urban citizens	In-depth interviews	Cow-calf separation; zero-grazing
Danne and Mushoff, 2017 [3]	Germany	Dairy farmers	Experiment	Pasture grazing
Hötzel et al., 2017 [77]	Brazil	Citizens	Self-administered survey	Zero-grazing, cow-calf separation
Ly et al., 2021 [17]	Canada and US	Citizens	Online survey	Cow-calf separation, excess dairy calves, pasture access
Naspetti et al., 2017 [85]	Austria, Belgium, Denmark, Finland, Italy, UK	Dairy farmers	Online survey, face-to-face, telephone options	Cow-calf separation
Neave et al., 2022 [34]	New Zealand	Dairy farmers	Telephone interview, semi-structured interview	Cow-calf contact
Palczynski et al., 2021 [86]	England	Dairy farmers (and other stakeholders)	In-depth, face-to-face, semi-structured interviews	Calf housing (and other aspects of calf disease management).
Perttu et al., 2020 [87]	North America (Minnesota)	Public	In-person survey	Dairy calf housing
Schuppli et al., 2014 [72]	US/Canada	Public [(un)affiliated with the dairy industry]	Online web forum	Pasture access
Smid et al., 2021 [81]	Canada (4 Western provinces)	Dairy farmers	Focus groups and semi-structured interviews	Outdoor access
Ventura et al. 2013 [9]	North America/Canada	Public, dairy industry conference attendees	Web-based forum	Cow-calf separation
Weinrich et al., 2020 [75]	Germany	Consumers	Survey	Housing/pasture access

**Table 3:** Overview of primary research on consumer-citizen and farmer perspectives on selected on-farm dairy technologies (milking related).

Citation	Country	Respondents	Method	Technological Focus
Driessen and Heutinck, 2015 [99]	The Netherlands	Farmers	Discourse analysis of grey literature, farmer interviews, observation	Automatic milking machines
Garguilo et al., 2018 [68]	Australia	Dairy Farmers (and input suppliers)	Online survey	Precision technologies
Hansen, 2015 [100]	Norway	Dairy farmers	In-depth interviews	Robotic milking machines
Holloway et al., 2014 [110]	UK	Dairy farmers	In depth interviews and observational studies	Robotic milking machines
Krampe et al., 2021 [35]	Finland, the Netherlands, Spain	Consumer	Focus groups	Precision livestock farming
Millar et al., 2002 [113]	UK	Consumers	Postal survey	AMS
Pfeiffer et al., 2020 [114]	Germany	Public	Online consumer survey	Digital farming technologies
Schewe and Stuart, 2015 [106]	US (Mid-west), the Netherlands, Denmark	Dairy farmers	Interviews	Automatic milking systems
Silvi et al., 2021 [115]	Brazil	Farmers	Online survey (via google forms)	Precision technologies (incl. milking robots, automated calf feeders)

## Data Availability

Not applicable.
